# Emergence and Enhancement of Ultrasensitivity through Posttranslational Modulation of Protein Stability

**DOI:** 10.3390/biom11111741

**Published:** 2021-11-22

**Authors:** Carla M. Kumbale, Eberhard O. Voit, Qiang Zhang

**Affiliations:** 1 Department of Biomedical Engineering, Georgia Institute of Technology and Emory University, 950 Atlantic Drive, Atlanta, GA 30332, USA; ckumbale3@gatech.edu; 2 Gangarosa Department of Environmental Health, Rollins School of Public Health, Emory University, 1518 Clifton Rd, Atlanta, GA 30322, USA

**Keywords:** ultrasensitivity, posttranslational modification, covalent modification cycle, protein stability, signal amplification

## Abstract

Signal amplification in biomolecular networks converts a linear input to a steeply sigmoid output and is central to a number of cellular functions including proliferation, differentiation, homeostasis, adaptation, and biological rhythms. One canonical signal amplifying motif is zero-order ultrasensitivity that is mediated through the posttranslational modification (PTM) cycle of signaling proteins. The functionality of this signaling motif has been examined conventionally by supposing that the total amount of the protein substrates remains constant, as by the classical Koshland–Goldbeter model. However, covalent modification of signaling proteins often results in changes in their stability, which affects the abundance of the protein substrates. Here, we use mathematical models to explore the signal amplification properties in such scenarios and report some novel aspects. Our analyses indicate that PTM-induced protein stabilization brings the enzymes closer to saturation. As a result, ultrasensitivity may emerge or is greatly enhanced, with a steeper sigmoidal response, higher magnitude, and generally longer response time. In cases where PTM destabilizes the protein, ultrasensitivity can be regained through changes in the activities of the involved enzymes or from increased protein synthesis. Importantly, ultrasensitivity is not limited to modified or unmodified protein substrates—when protein turnover is considered, the total free protein substrate can also exhibit ultrasensitivity under several conditions. When full enzymatic reactions are used instead of Michaelis–Menten kinetics for the modeling, the total free protein substrate can even exhibit nonmonotonic dose–response patterns. It is conceivable that cells use inducible protein stabilization as a strategy in the signaling network to boost signal amplification while saving energy by keeping the protein substrate levels low at basal conditions.

## 1. Introduction

### 1.1. Regulation of Protein Stability through Posttranslational Modifications

**Figure 1:**
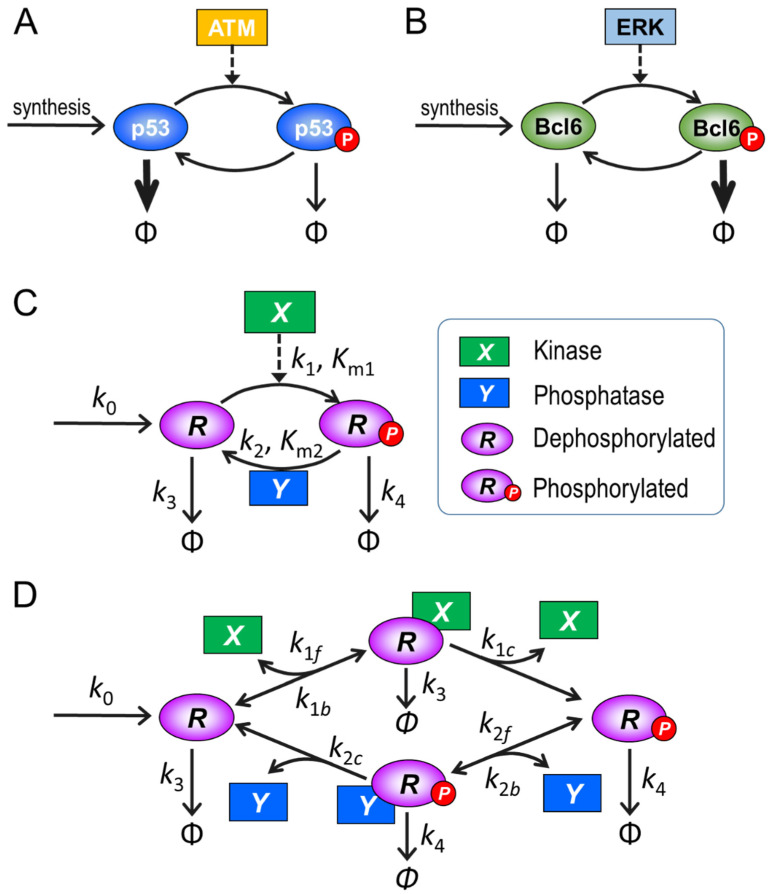
Schematic illustration of covalent modification cycles (CMCs) that result in altered protein stability and structures of computational models used here. (**A**) p53 stabilization by ATM-catalyzed phosphorylation. (**B**) BCL6 destabilization by ERK-catalyzed phosphorylation. (**C**) Generic model based on the phosphorylation–dephosphorylation cycle using Michaelis–Menten kinetics (MM model) and (**D**) generic model using mass-action kinetics (“full model”). Open arrow heads: mass fluxes, with thickness representing magnitude; dashed arrows with solid arrow heads: enzymatic catalysis.

It has been known for some time that posttranslational modifications (PTMs) are important mechanisms for regulating not only the activity of a protein, but also the abundance of a protein by means of changing its stability. A well-studied example is the DNA damage response. Once the tumor suppressor p53 is phosphorylated by upstream kinases, such as ATM (ataxia telangiectasia mutated), in response to DNA double-strand breaks, its half-life increases dramatically from less than 30 min to over 3 h (Figure 1A), which causes the accumulation of p53 that can induce target gene expression [1,2]. A second example, in some sense of the opposite nature, occurs in the germinal center response of B lymphocytes. B cell receptor-activated MAPK phosphorylates BCL6 (B-cell lymphoma 6), resulting in accelerated degradation of BCL6 by the ubiquitin/proteasome pathway (Figure 1B), which helps the B cells exit the germinal center response [3]. Many similar examples have been reported where protein stabilization or destabilization drives signaling, including IKK-mediated phosphorylation and degradation of IκB in the inflammatory response, Chk1-mediated phosphorylation and proteasomal degradation of Cdc25A during cell cycle arrest, and stabilization of ΔFosB by casein kinase 2-mediated phosphorylation, which might be responsible for long-term adaptation in the brain [4,5,6]. It is thus conceivable—and even likely—that altering protein stability and/or activity through the same PTM event may be an important controllable mode of dual regulation of cellular signaling networks in general. Expressed differently, if the abundance of a protein substrate can be fine-tuned through changes in protein stability, then these changes can in turn be used by the cell as modulators of both the dynamic and steady-state input-output (I/O) behaviors of covalent modification cycles (CMCs), which may or may not alter the activity of the protein.

### 1.2. Ultrasensitivity

Cell signaling networks display “ultrasensitivity” if small changes in input are amplified into much larger percentage changes in output [7,8]. An ultrasensitive I/O relationship is generally sigmoidal in shape and often approximated by a Hill function; the terminology suggests that an ultrasensitive response is steeper than the well-known hyperbolic trend of a Michaelian function [9,10]. Embedded in complex network structures such as feedback and feedforward loops, signal amplification is required for cells and organisms to achieve higher-order functions, including differentiation, proliferation, homeostasis, adaptation, and biological rhythms [11,12]. At least six major ultrasensitive response motifs (URM) have been identified in intracellular molecular networks, namely: (i) positive cooperative binding; (ii) homo-multimerization; (iii) multistep signaling; (iv) molecular titration; (v) zero-order CMCs; and (vi) positive feedback [12,13,14]. Each of these URMs has its own unique mechanism achieving signal amplification.

### 1.3. Ultrasensitivity through Zero-Order Covalent Modification Cycle

The ubiquitous zero-order CMC is particularly interesting, as it can generate nearly switch-like responses. A typical implementation is a modifying/demodifying cycle that is driven by PTMs involving phosphorylation, acetylation, oxidation, methylation, or sumoylation [15]. Specifically, protein activities can be regulated through covalent bonding of moieties to certain amino acid residues, such as phosphate to serine, threonine, and tyrosine in the case of phosphorylation, and an acetyl group to lysine in the case of acetylation. The local electrical charge, possibly accompanied by steric changes introduced by these moieties, can greatly affect the protein molecule’s interaction with other large or small molecules, thereby turning on or off the activity of the protein as an enzyme, transcription factor, or signaling molecule. Covalent modifications of proteins often require specific enzymes, such as kinases, acetyltransferases, methyltransferases, and oxidases, as well as counteracting (demodification) enzymes catalyzing the reverse reactions, such as phosphatases, deacetylases, demethylases, and reductases.

Signal amplification through CMCs was first predicted and analyzed with a mathematical model by Goldbeter and Koshland Jr. in the early 1980s [16,17]. It occurs when the two opposing enzymes driving the modification cycle of a protein are operating near saturation. In a phosphorylation–dephosphorylation cycle, for example, zero-order ultrasensitivity arises when the amount of protein substrate is at a concentration high enough to saturate the available kinase and phosphatase. Here, the terminology “protein substrate” is used to distinguish this protein from the involved enzymes. Under these conditions, small changes in the amount or activity of either the kinase or phosphatase can dramatically change the steady-state fraction of the amounts of phosphorylated or dephosphorylated substrates. Since the theoretical predictions by the Goldbeter–Koshland model, zero-order ultrasensitivity via covalent modification has been reported in numerous biological settings, in both prokaryotes and eukaryotes [18,19,20,21,22,23].

### 1.4. Caveat of the Goldbeter–Koshland Model Suggests a Mechanism of Signaling Control

One important conceptual simplification of the original Goldbeter–Koshland model is that the total abundance of the protein substrate in the CMC is regarded as constant, which ignores turnover via de novo protein synthesis and degradation. This omission is possibly critical in the context of protein signaling, as proteins are constantly synthesized and degraded. The assumption of constancy may largely be valid when the signaling events driven by PTM occur rapidly in comparison to the protein substrate turnover. However, even if signaling is fast, it is possible—and indeed a frequent observation as mentioned before—that the PTM alters the stability of the protein substrate, which secondarily affects the total amount of the protein substrate. We first reported that, due to the “leakiness” caused by protein turnover, zero-order ultrasensitivity is compromised when turnover is present, and that the steepness of the sigmoidal response deteriorates as the overall protein turnover rate increases [24]. More recently, Mallela et al. further elaborated on the importance of protein synthesis and turnover in affecting zero-order ultrasensitivity of CMCs, especially in the context of multiple PTM cascades sharing the same E3 ligase responsible for protein degradation [25]. Thus, the formerly simple results described by Goldbeter and Koshland are in truth more complicated, as they depend on the kinetic features of the involved enzymes, their saturation, and the degree of protein synthesis and turnover.

Here, we pursue the question how cells may use alternate PTM-induced changes in protein stability as an additional layer of control to modulate the zero-order ultrasensitive response of a CMC. In particular, we ask whether such modulations are sufficient to render or enhance ultrasensitivity by stabilizing the protein substrate, or diminish or destroy it by destabilizing the protein substrate. To answer these questions, we systematically study the governing kinetic features of the protein cycle one by one, with mathematical modeling, which allows us to modify many aspects or combinations of aspects of a protein signaling cycle with full knowledge of the system features and behaviors. We demonstrate that ultrasensitivity can be gained, enhanced, or attenuated for the modified, unmodified, and total protein substrates depending on the conditions affecting stability.

## 2. Methods

### 2.1. Model Structure and Parameterization

Our goal is to explore how the behavior of a CMC is affected if protein turnover, protein stability, and kinetic features of the governing enzymes are explicitly taken into account. For this exploration, we consider the generic signaling motif of a protein phosphorylation–dephosphorylation cycle (Figure 1C) as an “order-of-magnitude” model, i.e., a numerical model without absolutely precise determination of parameter values and with an expectation of qualitative, rather than quantitative results.

We started with a simple model consisting of two ordinary differential equations (ODEs), formulated in the tradition of mass-action and Michaelis–Menten (MM) kinetics:

(1) $$\frac{dR}{dt}=k_{0}-k_{1}\frac{X\times R}{\left(K_{m1}+R\right)}+k_{2}\frac{Y\times R_{p}}{\left(K_{m2}+R_{p}\right)}-k_{3}\times R,$$

(2) $$\frac{dR_{p}}{dt}=k_{1}\frac{X\times R}{\left(K_{m1}+R\right)}-k_{2}\frac{Y\times R_{p}}{\left(K_{m2}+R_{p}\right)}-k_{4}\times R_{p}$$

In the following, this model is referred to as the MM model. *R* is the protein substrate that is newly synthesized with rate *k*_0_. It can either be phosphorylated into *R_p_* by a kinase *X* which, as a default, follows typical MM kinetics with Michaelis constant *K_m_*_1_ and maximal velocity *V_max_*_1_ = *k*_1_*X*, or it can be degraded with a first-order rate constant *k*_3_. Analogously, *R_p_* can be dephosphorylated by a phosphatase *Y* that follows MM kinetics with a Michaelis constant *K_m_*_2_ and maximal velocity *V_max_*_2_ = *k*_2_*Y*. *R_p_* can also be degraded, in this case with a first-order rate constant *k*_4_.

Default parameter values are presented in Table 1. Since covalent protein modifications such as phosphorylation and dephosphorylation occur rapidly, at the order of seconds to minutes, while protein degradation occurs at a much slower rate, often with half-lives at the order of hours, the time scales between these two types of processes are often clearly separated by two or more orders of magnitude. Accordingly, we set default values for *k*_3_ and *k*_4_ to be 1/100 of *k*_1_/*K_m_*_1_ and *k*_2_/*K_m_*_2_, respectively, because these two ratios approximate the apparent first-order time constants at which phosphorylation and dephosphorylation occur when the kinase and phosphatase are far from saturation. Unless otherwise specified, *Y* is kept constant with value 1.

It is well known that the MM kinetics is a simplification that approximates the full mass-action enzymatic kinetics with certain constrains [26]. These constraints include that the substrate exists in excess of the enzyme and the substrate–enzyme complex quickly reaches a quasi-steady state. In many cellular circumstances, some of the assumptions may not be valid. An example is the case where the substrate and enzyme are proteins with comparable concentrations [27,28]. Under conditions where the assumptions are not satisfied, the full kinetic model should be utilized. Indeed, it has been shown that implementing the full model can generate nonlinear dynamics that may be missed by simple MM kinetics [26,29]. To assess the role of these differences, we used the full kinetic model (Figure 1D and Table 2) to validate or refute the findings based on the approximating MM model. The ODEs for the full model are provided in the Supplemental Material. In this full model, we assume that *R* or *R_p_*, complexed with their respective enzymes *X* or *Y*, can still be independently degraded like the respective free forms and with the same half-lives, and when they are destroyed, *X* and *Y* are released and recycled. That a protein component in a multimeric protein complex can be specifically targeted and degraded by the ubiquitination/proteasomal pathway has been well established [30,31,32]. This matter is discussed further in the Discussion section.

### 2.2. Metrics of Ultrasensitivity

Simulations start with the initial values of variables equal to the respective steady-state concentrations, which are achieved when *X* = 0 for the MM model or *X_tot_* = 0 for the full model, where the initial values of *R_p_*, *RX*, and *R_p_Y* are always zero. For fair comparisons, all dose–response (DR) curves are obtained once the model has achieved steady state. The increment size of the input level of *X* is 1% within the dose range indicated. The degree of ultrasensitivity of a steady-state DR curve can be evaluated with two related metrics that are commonly used in the field. First, the Hill coefficient, *n_H_*, may be approximated from the equation

(3) $$n_{H}=\frac{\ln81}{\ln\frac{X_{0.9}}{X_{0.1}}}$$

where *X*_0.9_ and *X*_0.1_ are the concentrations of *X* that produce 90% and 10%, respectively, of the maximal response (after subtracting the background response level when *X* = 0) [12]. *n_H_* represents the overall steepness or global degree of ultrasensitivity of the DR curve. Second, we evaluate the local response coefficient (*LRC*) of a DR curve by calculating all slopes of the curve on dual-log scales, which are equivalent to the ratios of the fractional change in response (*R*) to the fractional change in dose (*D*) [7]:

(4) $$LRC=\frac{\mathrm{dln}R}{\mathrm{dln}D}$$

The maximal |*LRC*| of a DR curve (|*LRC*|*_max_*) represents the maximal amplification capacity of the signaling motif. The comparison between *n_H_* and *LRC* is important as these quantities are not necessarily equivalent and they vary depending on the basal response level and the shape of the DR curve. Typical ultrasensitive responses have |*n_H_*| and |*LRC*|*_max_* values substantially above 1. However, if a DR curve is very sigmoidal with high |*n_H_*|, but its |*LRC*|*_max_* < 1, then there is no signal amplification, and higher-order cellular functions such as bistability, robust adaptation, and oscillation cannot be achieved [12,33,34]. Thus, *n_H_* alone can misrepresent the actual degree of signal amplification and must be cross-examined with *LRC*.

### 2.3. Simulation Tools

The models were coded in MATLAB (MathWorks, Natick, MA, USA) and all simulations were run with differential equation solver ode15s. The model codes in MATLAB format as well as in R format are available as additional Supplemental files and can also be accessed at the GitHub repository https://github.com/pulsatility/Effects-of-Protein-Stability-on-Ultrasensitivity.git, accessed on: 17 November 2021.

## 3. Results

### 3.1. Ultrasensitivity in the Absence of PTM-Induced Changes in Protein Stability

**Figure 2:**
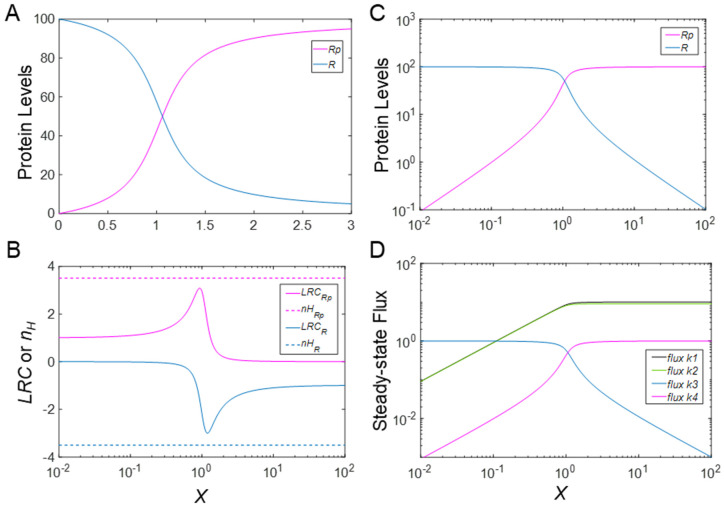
Steady−state does−response (DR) curves of *R* and *R_p_*, associated fluxes, *n_H_* and *LCR*, as functions of kinase *X* activity in the MM model. (**A**) DR curves of *R* vs. *X* and *R_p_* vs. *X* on linear scale. (**B**) *n_H_* and *LRC* of DR curves of *R* vs. *X* and *R_p_* vs. *X*. (**C**) DR curves of *R* vs. *X* and *R_p_* vs. *X* in a log-log representation. (**D**) Fluxes, named by associated rate constant, plotted against *X*. Specifically, *flux k*_1_: phosphorylation; *flux k*_2_: dephosphorylation; *flux k*_3_: degradation of *R*; and *flux k*_4_: degradation of *R_p_*.

To create a baseline, we start with the default setting *k*_3_ = *k*_4_ = 0.01, which reflects that the phosphorylation status of *R* does not affect its stability. As a consequence, the total steady-state protein substrate concentration *R_tot_* (=*R* + *R_p_*) remains constant even if the activity of the kinase *X* varies. Additionally, the *k*_3_ and *k*_4_ values are very small in comparison to *k*_1_ and *k*_2_. Since *R_tot_* typically exceeds *K_m_*_1_ and *K_m_*_2_ by 10-fold or more, and as expected for the CMC motif, the steady-state DR curves of *R* vs. *X* and *R_p_* vs. *X* are sigmoidal on the linear scale (Figure 2A) with *n_H_* at −3.51 and 3.51, respectively (Figure 2B); the negative sign for *n_H_* indicates a decreasing or inhibitory response. On a log scale, the quasi-exponential rise in *R_p_* and decay of *R* flatten toward straight lines (Figure 2C).

The degree of local ultrasensitivity, as measured by *LRC*, varies across the range of *X* and peaks in the center of the DR curves at approximately −3.0 and +3.1 for *R* and *R_p_*, respectively (Figure 2B). Thus, |*n_H_*| in this case is a slight overestimate of the corresponding |*LRC*|*_max_*. The phosphorylation and dephosphorylation fluxes (with rates *k*_1_ and *k*_2_, respectively) are dominant over the relatively small protein turnover fluxes with rates *k*_3_ and *k*_4_ at the steady state for large input values of X (Figure 2D). In a logarithmic representation, these MM fluxes increase essentially linearly as *X* increases before approaching plateaus (Figure 2D). When protein production and degradation are considered negligible, by setting *k*_0_, *k*_3_ and *k*_4_ to zero, the ultrasensitive responses are slightly enhanced, and the Hill coefficients and (|*LRC*|*_max_* rise in magnitude to 3.74 and 3.45 for *R_p_* and −3.74 and −3.34 for *R* (simulation results not shown).

### 3.2. Effects of Protein Stability on Ultrasensitivity

In this section, we suppose that changes in the stability of *R_p_* can be introduced by the PTM, and thus by means of the kinase *X*, as it has been observed numerous times [1,3,4,5,6]. When the protein substrate *R* is phosphorylated to *R_p_*, the stability of *R_p_* is affected, which translates into an increasing or decreasing rate of degradation, *k*_4_. In particular, if the PTM stabilizes *R_p_*, i.e., *k*_4_ decreases, the amount of *R_p_* increases, and *R_tot_* is expected to increase accordingly. This rise in *R_tot_* secondarily alters the degree of saturation of the phosphorylation and dephosphorylation reactions and, consequently, is expected to affect the degree of ultrasensitivity in the DR curves. These overall effects could theoretically also be caused by changes in *k*_3_, but we focus on *k*_4_ because the PTM directly affects the stability of *R_p_*, whereas *R* is affected only in a secondary manner.

#### 3.2.1. Effects on Steady-State *R*

**Figure 3:**
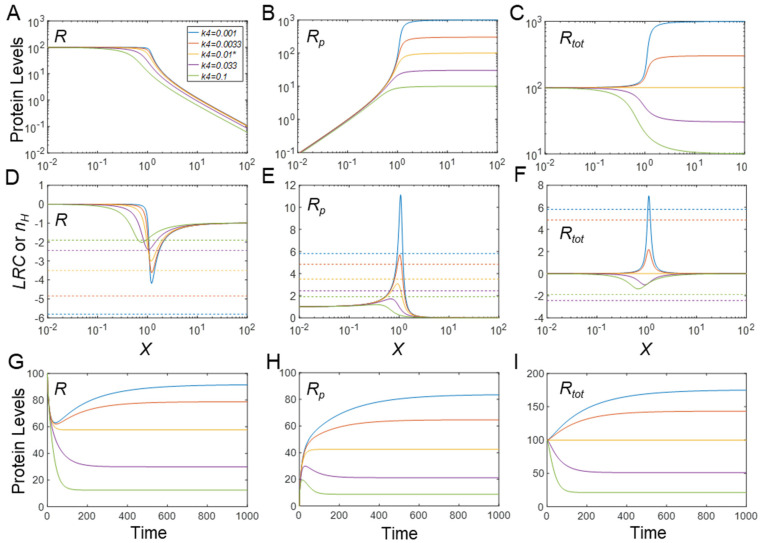
Effects of phosphorylation−induced changes in protein stability on ultrasensitivity and response time in the MM model. (**A**–**C**) Steady-state DR curves for *R* vs. *X*, *R_p_* vs. *X*, and *R_tot_* vs. *X*, respectively, for different values of *k*_4_, as indicated in panel (**A**). The same color scheme for *k*_4_ values holds for all panels. (**D**–**F**) *LRC* (solid lines) and *n_H_* (dashed horizontal lines) pertain to *R*, *R_p_*, and *R_tot_*, respectively. (**G**–**I**) Response of *R*, *R_p_*, and *R_tot_* over time, induced by *X* = 1, respectively. * *k*_4_ = 0.01 is the default value.

When the PTM increases the stability of *R_p_*, i.e., *k*_4_ decreases, the steady−state DR curves of *R* vs. *X* (Figure 3A) and *R_p_* vs. *X* (Figure 3B) both become steeper; conversely, when the stability of *R_p_* decreases, i.e., *k*_4_ increases, the two curves become shallower. The changes in the steepness of the DR curves can be quantified by *n_H_* as well as the maximal local ultrasensitivity, |*LRC*|*_max_*. Both increase as *k*_4_ decreases (Figure 3D,E). Interestingly, however, for *k*_4_ values comparable to or below the default value, |*LRC*|*_max_* is generally lower than |*n_H_*| for the *R* vs. *X* response, which is an indication that the Hill coefficient overestimates the maximal degree of signal amplification in these situations (Figure 3D). For *k*_4_ values rising above the default value, |*LRC*|*_max_* starts to match up with |*n_H_*| and eventually exceeds it. For very large *k*_4_ values, |*n_H_*| approaches a constant value of approximately 1.58 and |*LRC*|*_max_* approaches a constant value of approximately 1.72. Thus, there is still ultrasensitivity, but its degree is modest. The value of the kinase activity *X* at which |*LRC*| is maximal shifts to the left as *k*_4_ increases.

#### 3.2.2. Effects on Steady-State *R_p_*

The elevated steepness of the *R_p_* vs. *X* response, with increased stability of *R_p_*, is evidently due to the increasing maximal *R_p_* level when *k*_4_ decreases (Figure 3B). Interestingly, and contrary to the effect on the response of *R* vs. *X*, *LRC_max_* is generally higher than *n_H_* for *k*_4_ values below the default value, indicating that the Hill coefficient is underestimating the maximal degree of signal amplification (Figure 3E). For *k*_4_ values higher than the default value, *LRC_max_* starts to match *n_H_* and eventually drops below its value. For very large *k*_4_ values, *n_H_* approaches 1.58, whereas *LRC*_max_ settles at approximately 1. The value of kinase activity *X* for which *LRC* is maximal shifts to the left as *k*_4_ increases.

#### 3.2.3. Effects on Steady-State *R_tot_*

In the Goldbeter–Koshland model of the CMC, either *R* or *R_p_* is regarded as the output, because the activities of either one may change by the phosphorylation status. However, in some situations, the covalent modification status of an amino acid residue may only affect protein stability without affecting protein activity [35,36]. In these cases, *R_tot_* should be viewed as the output. Depending on the values of *k*_4_, the response of *R_tot_* vs. *X* can be either stimulatory or inhibitory (Figure 3C), because either more or less *R_p_* is removed from the system. At the default level of *k*_4_, which is equal to *k*_3_, *R_tot_* does not change with *X*. However, as the PTM stabilizes *R_p_*, i.e., *k*_4_ decreases from the default value, the steady-state response of *R_tot_* vs. *X* increases monotonically to a higher plateau than before and also becomes increasingly steeper, with *LRC_max_* surpassing *n_H_* for very low *k*_4_ values (Figure 3F). Conversely, as the PTM destabilizes *R_p_*, the steady-state response of *R_tot_* vs. *X* decreases monotonically toward a lower plateau and also becomes increasingly more sigmoidal (despite that the response of *R_p_* itself is no longer ultrasensitive), with |*LRC*|*_max_* approaching 1.72 for very high *k*_4_ values. Surprisingly, |*n_H_*| changes in the opposite direction to |*LRC*|*_max_* for *k*_4_ values above the default value (Figure 3F). A small increase in *k*_4_ above the default value first results in a very high |*n_H_*|, but as *k*_4_ increases further, |*n_H_*| drops back and approaches 1.58. This inverse relationship between |*LRC*|*_max_* and |*n_H_*| demonstrates again that these two metrics do not always conform to each other and that reliance on the Hill coefficient as an estimate of the degree of signal amplification can be misleading. In summary, both stabilization and destabilization of *R_p_* can lead to the enhancement of ultrasensitivity in the steady-state response curve of *R_tot_* vs. *X*.

While *R_p_* and *R* are expected to exhibit ultrasensitivity due to the zero-order covalent modification effect, as revealed by the Goldbeter–Koshland model, it is interesting to note that *R_tot_* also exhibits various degrees of ultrasensitivity depending on the value of *k*_4_, i.e., the stability of *R_p_*. To dissect this mechanism leading to ultrasensitivity for *R_tot_*, we use the following two steady-state flux and mass conservation equations to solve for *R_tot_*:

(5) $$k_{0}=k_{3}R+k_{4}R_{P}$$

(6) $$R_{tot}=R+R_{p}$$

By substituting either *R* or *R_p_* from Equation (5) in Equation (6), we obtain two equations that exhibit symmetry with respect to *k*_3_ and *k*_4_, namely

(7) $$R_{tot}=\frac{k_{0}}{k_{3}}+\left(1-\frac{k_{4}}{k_{3}}\right)R_{P}$$

(8) $$R_{tot}=\frac{k_{0}}{k_{4}}+\left(1-\frac{k_{3}}{k_{4}}\right)R$$

The equations say that except for cases where *k*_3_ and *k*_4_ are equal, the steady-state *R_tot_* scales linearly with both *R_p_* or *R*. When *k_3_* > *k_4_*, i.e., phosphorylation results in *R_p_* stabilization, *R_tot_* has a basal level determined by *k_0_*/*k_3_* and increases as *R_p_* increases (Equation (7)). For very small *k*_4_, *R_tot_* ≈ *k*_0_/*k*_3_ + *R_p_*. Since the response curve *R_p_* vs. *X* is always monotonically increasing (Figure 3B), its ultrasensitivity is passed to *R_tot_* with comparable *n_H_* values. By contrast, the *LRC* of the *R_tot_* response will be lower than that of the *R_p_* response due to the presence of the basal level at *k*_0_/*k*_3_ (Figure 3E vs. Figure 3F). Conversely, if phosphorylation results in *R_p_* destabilization, i.e., *k_3_* < *k_4_*, *R_tot_* has a minimal level determined by *k*_0_/*k*_4_ (Equation (8)). For very large *k_4_*, *R_tot_* ≈ *k*_0_/*k*_4_ + *R*. Since the response curve of *R* vs. *X* is always monotonically decreasing (Figure 3A), its ultrasensitivity is passed to *R_tot_* with comparable *n_H_* values, and again, the |*LRC*| of the *R_tot_* response is lower than that of the *R* response, due to the presence of the minimal level *k*_0_/*k*_4_ (Figure 3D vs. Figure 3F).

#### 3.2.4. Effects on Timing of Signaling

PTMs can have an effect on the timing of signaling. When they induce changes in protein stability, the time it takes the signaling motif to reach steady state in response to X is no longer determined only by the covalent modification reactions, but also by the half-lives of the protein substrate. Not surprisingly, for *k*_4_ lower than the default value, it takes much longer time for *R*, *R_p_* and *R_tot_* to reach their steady state (Figure 3G–I). The trajectory of *R* is nonmonotonic—it first decreases quickly as a result of the phosphorylation of pre-existing *R* and then rises slowly (because *R_tot_* increases) to settle at a new steady state (Figure 3G). In comparison, *R_p_* first shoots up quickly as a result of the phosphorylation of pre-existing *R* into *R_p_*, and then rises slowly toward its new steady state (Figure 3H). *R_tot_* does not exhibit a biphasic trend and instead increases gradually toward its new steady state (Figure 3I). For *k*_4_ higher than the default value, the time it takes to reach the steady state does not appear to be monotonically correlated with *k*_4_ (Figure 3G–I). For *k*_4_ values slightly higher than *k*_3_, the differential stability of *R* and *R_p_* causes the system to approach the steady state slowly because the protein half-life, rather than the fast MM reactions, dominates the long-term kinetics (Figure 3G,H, purple vs. orange lines). However, as *k*_4_ increases further, the responses are overall faster since the overall protein half-life becomes shorter (Figure 3G–I, green vs. purple lines). Generally, *R* first decreases quickly as a result of phosphorylation of pre-existing *R* and then continues to decrease till it settles to a new steady state (Figure 3G). In comparison, *R_p_* exhibits a nonmonotonic trajectory—it first rises quickly as a result of phosphorylation of pre-existing *R* into *R_p_*, and then decreases (because *R_tot_* decreases) slowly to settle at a new steady state (Figure 3H). *R_tot_* has a similar monotonically decreasing profile as *R* (Figure 3I).

#### 3.2.5. Behaviors of the Full Model

**Figure 4:**
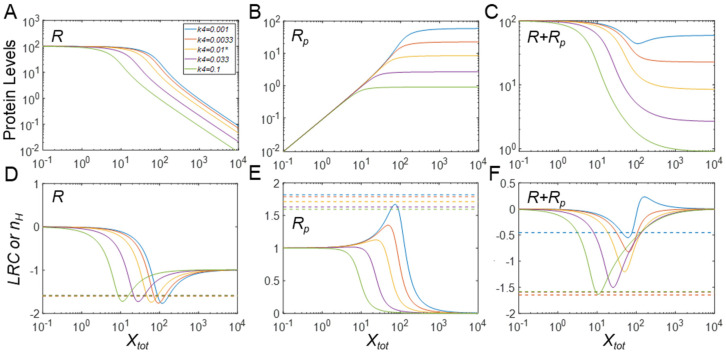
Effects of phosphorylation-induced changes in protein stability on ultrasensitivity in the full model when enzyme and basal substrate levels are comparable. (**A**–**C**) Steady-state DR curves for *R* vs. *X_tot_*, *R_p_* vs. *X_tot_*, and free *R_tot_* (*R* + *R_p_*) vs. *X_tot_*, respectively, for different values of *k*_4_, as indicated in panel (**A**). The same color scheme for *k*_4_ values holds for all panels. (**D**–**F**) *LRC* (solid lines) and *n_H_* (dashed horizontal lines) pertain to *R*, *R_p_*, and free *R_tot_*, respectively. * *k*_4_ = 0.01 is the default value. For these simulations, *Y_tot_* was set to 100.

For the full model, the steady-state DR behavior of *R*, *R_p_* and free *R_tot_* (*R* + *R_p_*) with respect to *X_tot_* (the input parameter of the full model) and their dynamical responses are nearly identical to the simple MM model (simulation results not shown), indicating that the enhancement of ultrasensitivity by protein stabilization is a robust feature that is insensitive to implementation details under the current parameter conditions where the protein substrate is in excess of the enzymes (i.e., for the default setting *R_tot_* = 100 vs. *Y_tot_* = 1). While such strong differences in molecular abundances may be encountered in cell signaling and metabolic pathways, this situation is not general. Instead, the covalent modification enzymes may be at comparable levels to their protein substrates including those involved in the MAPK signaling cascade [27,28], suggesting for simulation use of the full model rather than the simplifying MM model. In particular, the apparent Michaelis constants *K_m_*_1_ and *K_m_*_2_ are substituted with their original definitions as (*k*_1*b*_ + *k*_1*c*_)/*k*_1*f*_ and (*k*_2*b*_ + *k*_2*c*_)/*k*_2*f*_, respectively. The consequence of increasing the enzyme level is shown in Figure 4: increasing *Y_tot_* to 100, up to a point where *Y_tot_* = *R_tot_*, markedly weakens the degree of ultrasensitivity. Absent PTM-induced changes in *R_p_* stability, both *R* and *R_p_* are now only marginally ultrasensitive in response to *X_tot_* (Figure 4A,B,D,E). However, when stabilization of *R_p_* occurs with lower *k*_4_ values, the ultrasensitivity of *R_p_* is enhanced to some extent while the ultrasensitivity of *R* remains basically unchanged. Compared with the MM model (Figure 3 B,E), the enhancement of the ultrasensitivity of *R_p_* in the full model is not as strong. The reason is that the increase in the total protein substrate abundance, which can be achieved due to stabilization of *R_p_* in the full model, is not as high as can be achieved in the MM model. In the full model, a significant amount of *R* is titrated in the complex with enzyme *X* and thus not stabilized. As a result, the total protein substrate, including free substrates and substrates complexed with the two enzymes, only approaches 2.3-fold of the basal level when *k*_4_ is decreased by 10-fold from the default value (results not shown), in contrast to the 10-fold increase in the MM model (Figure 3C). Interestingly, when free *R_tot_* (*R + R_p_*) is considered as the model output, it exhibits a monotonically decreasing but slightly ultrasensitive DR relationship at the default *k*_4_ value (Figure 4C). This outcome occurs because of sequestration of *R and R_p_* by the enzymes *X* and *Y*, respectively, as *X_tot_* increases. More interestingly, with stabilization of *R_p_* where *k*_4_ value is further lowered, a nonmonotonic DR curve for free *R_tot_* emerges, which results from a rising total abundance of the protein substrate due to stabilization at higher *X_tot_* levels. When destabilization of *R_p_* occurs, the DR profile of free *R_tot_* follows *R*, the dominant free form, which is monotonically decreasing.

### 3.3. Protein Stabilization Can Lead to Emergence of Ultrasensitivity

**Figure 5:**
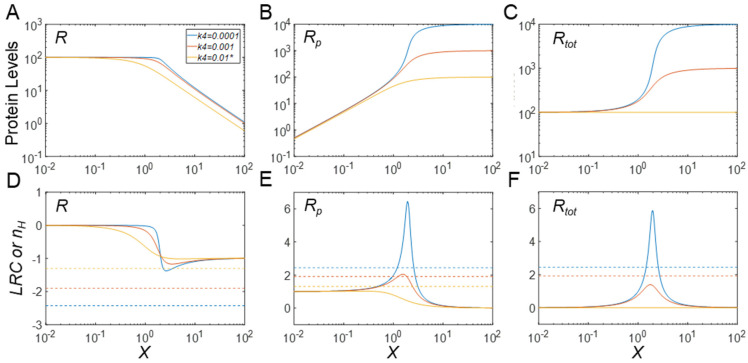
Emergence of ultrasensitivity through phosphorylation-induced protein stabilization in the MM model. (**A**–**C**) Steady−state DR curves for *R* vs. *X*, *R_p_* vs. *X*, and *R_tot_* vs. *X*, respectively, for different values of *k*_4_, as indicated in panel A. The same color scheme for *k*_4_ values holds for all panels. As *k*_4_ decreases, ultrasensitivity emerges for *R_p_* and *R_tot_*. (**D**–**F**) *LRC* (solid lines) and *n_H_* (dashed horizontal lines) for *R*, *Rp*, and *R_tot_*, respectively, for different values of *k*_4_. * *k*_4_ = 0.01 is the default value. For these simulations, the Michaelis constants were set to *K_m_*_1_ = *K_m_*_2_ = 100.

As we demonstrated for a CMC with pre-existing ultrasensitivity, stabilization of *R_p_* can enhance the degree of ultrasensitivity of the responses. In this section, we explore the possibility that stabilization of *R_p_* can render a formerly non-ultrasensitive CMC ultrasensitive. To demonstrate this possibility, we first eliminate ultrasensitivity by raising the default values of the Michaelis constants 10-fold, such that in the MM model *K_m_*_1_ = *K_m_*_2_ = 100, a value that is the same as the substrate *R_tot_* level with the default value of *k*_4_ at 0.01. As a result, the cycle no longer exhibits ultrasensitivity (Figure 5A–C), as evaluated by |*LRC*|*_max_* (Figure 5D–F). Starting with this new baseline, we now let *k*_4_ decrease below 0.01, which causes *R_p_* to be more stable than *R*. Indeed, the responses, especially the steady-state DR curves for *R_p_* vs. *X* and *R_tot_* vs. *X*, all begin to show a trend toward ultrasensitivity, as the total protein substrate level approaches and eventually surpasses the Michaelis constants *K_m_*_1_ and *K_m_*_2_, thereby pushing the phosphorylation and dephosphorylation cycle toward saturation (Figure 5A–C). These results demonstrate that ultrasensitivity can emerge de novo with PTM-induced protein stabilization.

For comparison, we used the full model to validate the results of the simple MM model. Again, we eliminated ultrasensitivity by setting apparent *K_m_*_1_ = *K_m_*_2_ = 100, which was accomplished by increasing the values of both *k*_1*b*_ and *k*_2*b*_ to 990. With these settings, simulations show results that are nearly identical to those of the simple MM model; in particular, ultrasensitivity emerges for both free *R*_p_ and free *R_tot_* (Figure S1). We also explored the situation where ultrasensitivity is eliminated from the full model by increasing the phosphatase *Y_tot_* level. When *Y_tot_* is gradually increased to 300, the ultrasensitivity of *R_p_* basically disappears (Figure S2B). At this new baseline level, *Y_tot_* has a value 3 times the basal *R_tot_* level. If *k*_4_ is now decreased to mimic stabilization of *R_p_*, ultrasensitivity re-emerges for *R_p_*, albeit only marginally, as indicated by |*LRC*|*_max_* (Figure S2B). The *R* response is also ultrasensitive but the degree is not affected by *k*_4_ (Figure S2A). Free *R_tot_* exhibits a similar DR profile and ultrasensitivity as R (Figure S2C).

### 3.4. Regulation of Protein Modification Cycles through Alterations in Enzyme Features

**Figure 6:**
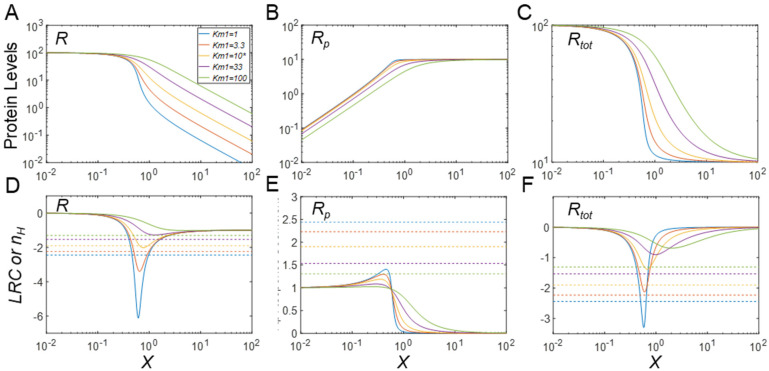
Effects of *K_m_*_1_ on ultrasensitivity under phosphorylation-induced protein destabilization in the MM model (*k*_4_ = 0.1). (**A**–**C**) Steady-state DR curves for *R* vs. *X*, *R_p_* vs. *X*, and *R_tot_* vs. *X*, respectively, for different values of *K_m_*_1_, as indicated in A. The same color scheme for *K_m_*_1_ values holds for all panels. The degree of ultrasensitivity increases for decreasing values of *K_m_*_1_. (**D**–**F**) *LRC* (solid lines) and *n_H_* (dashed horizontal lines) for *R*, *R_p_*, and *R_tot_*, respectively. * *K_m_*_1_ = 10 is the default value.

Given the important role of enzyme saturation by the substrate in CMC-mediated ultrasensitivity, we explore further in this section whether changes in the kinetic features of the modifying or demodifying enzymes can modulate the DR curves and their ultrasensitivity. Specifically, we investigate how changes in the Michaelis constants *K_m_*_1_ and *K_m_*_2_ modulate the steady-state DR curves and their ultrasensitivity. As a first example, we consider *K_m_*_1_ and examine the case where phosphorylation of *R* to *R_p_* results in destabilization, using as the baseline *k*_4_ = 0.1, which is 10-fold greater than *k*_3_. As *K_m_*_1_ decreases, the DR curves for *R* and *R_tot_* in the MM model become increasingly more sigmoidal (Figure 6A,C), with limited changes in the *R_p_* responses (Figure 6B). For low *K_m_*_1_ values, |*LRC*|*_max_* can be much greater than |*n_H_*|, whereas for high *K_m_*_1_ values, |*n_H_*| approaches 1.12, and |*LRC*|*_max_* approaches 1, indicating loss of ultrasensitivity (Figure 6A,D). For the *R_p_* response, increasing *K_m_*_1_ reduces the steepness of the DR curve with |*n_H_*| approaching 1.25, and ultrasensitivity is lost for high *K_m_*_1_ values as indicated by |*LRC*| below 1 (Figure 6B,E). Lastly, increasing *K_m_*_1_ reduces the steepness of the DR curve for *R_tot_* with |*n_H_*| approaching 1.25, and ultrasensitivity is lost for high *K_m_*_1_ values as indicated by |*LRC*| below 1 (Figure 6C,F). Varying the Michaelis constant *K_m_*_2_ of the phosphatase has a similar effect on ultrasensitivity (Figure S3). The full model produces very similar responses when the apparent *K_m_*_1_ is varied by either changing *k*_1*f*_ or *k*_1*b*_, even for the situation where the basal substrate and enzyme levels are comparable (e.g., *Y_tot_* = 100; simulation results not shown).

**Figure 7:**
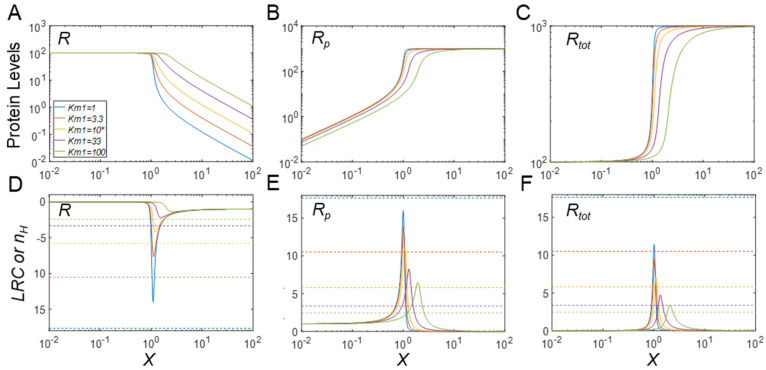
Effects of *K_m_*_1_ on ultrasensitivity under phosphorylation-induced protein stabilization in the MM model (*k*_4_ = 0.001). The results here pertain to a value of *k*_4_ that is 10-fold lower than the default. (**A**–**C**) Steady-state DR curves for *R* vs. *X*, *R_p_* vs. *X,* and *R_tot_* vs. *X*, respectively, for different values of *K_m_*_1_, as indicated in A. The same color scheme for *K_m_*_1_ values holds for all panels. (**D**–**F**) *LRC* (solid lines) and *n_H_* (dashed horizontal lines) for *R*, *R_p_*, and *R_tot_*, respectively. * *K_m_*_1_ = 10 is the default value.

The rationale for a second analysis is the situation where phosphorylation of R into R_p_ results in strong protein stabilization (*k*_4_ = 0.001, 10-fold lower than *k*_3_). When K_m1_ decreases below its baseline value of 10 in this situation in the MM model, the DR curves for *R*, *R_p_* and *R_tot_* become increasingly sigmoidal. For the response of *R*, |*n_H_*| obviously overestimates the degree of ultrasensitivity as evaluated by |*LRC*|_max_ (Figure 7A,D). By contrast, for high *K_m_*_1_ values, |*n_H_*| approaches 1.93, and |*LRC*|_max_ approaches 1, indicating loss of true ultrasensitivity. For the *R_p_* response, increasing *K_m_*_1_ reduces the steepness of the DR curve with |*n_H_*| approaching 2.61, and |*LRC*|_max_ is reduced to 4.97 with some, but not a complete loss of ultrasensitivity (Figure 7B,E). Except for very high *K_m_*_1_ values, |*LRC*|_max_ is generally higher than |*n_H_*|. The reason that large *K_m_*_1_ values do not result in complete loss of ultrasensitivity is that *K_m_*_2_ is still kept at default value of 10, thus keeping the dephosphorylation step close to saturable. Lastly, increasing *K_m_*_1_ reduces the steepness of the DR curve for *R_p_* with |*n_H_*| approaching 2.61, while |*LRC*|_max_ is reduced to 2.34 with some loss of ultrasensitivity (Figure 7C,F). Except for very low *K_m_*_1_ values, |*LRC*|_max_ is generally higher than |*n_H_*|. Varying *K_m_*_2_ has a similar effect on ultrasensitivity (Figure S4). The full model produces very similar responses when the apparent *K_m_*_1_ is varied by either changing *k*_1*f*_ or *k*_1*b*_ (simulation results not shown). For the condition when the basal substrate and enzyme levels are comparable by setting *Y_tot_* = 100, the degree of ultrasensitivity of the *R* and *R_p_* responses is generally lower, but still increases as the apparent *K_m_*_1_ decreases. However, similar to Figure 4C, nonmonotonic dose response emerges for free *R_tot_* (simulation results not shown). The response patterns of ultrasensitivity modulation by apparent *K_m_*_2_ are generally similar.

In addition, we studied the effects of changing the catalytic constant *k*_2_ (for the MM model) or *k*_2*c*_ (for the full model) of the phosphatase reaction on ultrasensitivity. In a nutshell, changes in *k*_2_ barely affect the degree of ultrasensitivity when *k*_3_ < *k*_4_ (Figure S5), ultrasensitivity increases considerably as *k*_2_ increases when *k*_3_ > *k*_4_ (Figure S6). Varying *k*_1_ (for the MM model) or *k*_1*c*_ (for the full model) merely shifts the DR curves horizontally without changing the degree of ultrasensitivity (simulation results not shown).

### 3.5. Ultrasensitivity in Response to Changes in Protein Synthesis Rate

**Figure 8:**
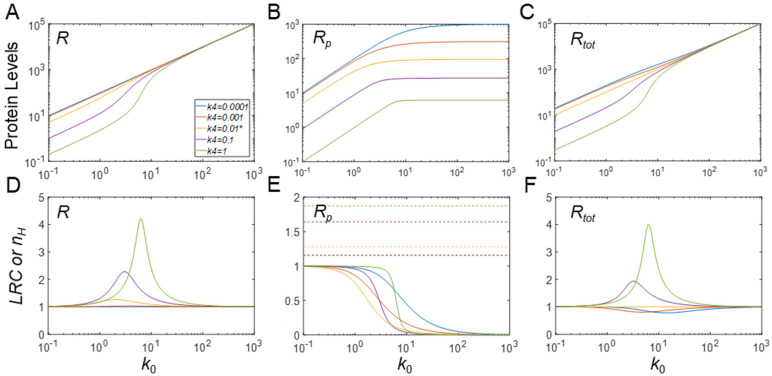
*k*_0_-driven ultrasensitivity with phosphorylation-induced changes in protein stability in the MM model. (**A**–**C**) Steady-state DR curves for *R* vs. *k*_0_, *R_p_ v.s k*_0_, and *R_tot_* vs. *k*_0_, respectively, for different values of *k*_4_, as indicated in (**A**). The same color scheme for *k*_4_ values is used for the other panels. (**D**–**F**) *LRC* (solid lines) and *n_H_* (dashed horizontal lines) for *R*, *R_p_*, and *R_tot_*. * *k*_4_ = 0.01 is the default value. *X* = 1 for all conditions. Note that no *n_H_* was evaluated for *R* and *R_tot_* because the responses do not saturate.

Lastly, we examined whether changes in the synthesis of *R* can lead to ultrasensitivity when PTM induces changes in protein stability. Suppose the kinase *X* displays an intermediate activity level of 1 matching the *Y* level, and the rate of synthesis of *R*, *k*_0_, is varied. Interestingly, when *R_p_* is destabilized in the MM model, i.e., *k*_4_ > *k*_3_, *R* and *R_tot_* at steady state exhibit ultrasensitive responses for a certain range of values of *k*_0_ even though their responses never plateau (Figure 8A,C). By contrast, if *k*_0_ is gradually increased, *R_p_* initially increases linearly (in log space), then plateaus, not exhibiting ultrasensitivity for any value of *k*_0_ (Figure 8B). When *k*_3_ = *k*_4_, *R_tot_* is proportional to *k*_0_, and *R* is slightly ultrasensitive. For stabilization of *R_p_*, and thus *k*_3_ > *k*_4_, the response of *R* vs. *k*_0_ is linear, while the response of *R_tot_* vs. *k*_0_ exhibits slight subsensitivity, with *LRC* dipping below 1 for some range of *k*_0_ (Figure 8F).

The emergence of ultrasensitivity in the responses of *R* and *R_tot_* for high *k*_4_ values may be counterintuitive, since destabilization of *R_p_* is believed to drive the enzymes away from saturation. The reason for ultrasensitivity to occur is the saturation of the flux through the phosphorylation (*k*_1_) step: when *k*_0_ approaches a high value like 10, any further small increase only leads to an increase in *R*, but not *R_p_*, and the result is ultrasensitivity. Actually, this mechanism of ultrasensitivity is a variant of zero-order degradation, which no longer requires the dephosphorylation reaction. By setting *k*_2_ = 0, i.e., disabling dephosphorylation, ultrasensitivity in the *R* and *R_tot_* responses remains strong (Figure S7).

With the default parameter values, the full model produces responses that are nearly identical to the results from the MM model (simulation results not shown). In comparison, when both *X_tot_* and *Y_tot_* are increased to 100, the ultrasensitivity of both *R* and free *R_tot_* (*R* + *R_p_*) in response to *k*_0_ is greatly enhanced, especially with high destabilization of *R_p_* when *k_4_* = 1, compared with the MM model (Figure S8A,C). This enhancement is likely due to the molecular titration effect by *X_tot_* and *Y_tot_*. However, when there is no change in stability of *R_p_*, or when it is stabilized, mild ultrasensitivity also occurs for *R* and free *R_tot_*, which is missing in the MM model. Moreover, *R_p_* also exhibits mild ultrasensitivity in response to *k*_0_, a feature that is missing in the MM model (Figure S8B). A summary of the main results is provided in Table 3.

## 4. Discussion

Cellular signal transduction pathways and gene regulatory networks regularly involve PTMs of protein components as a means of regulating their activities and abundances. Nearly all PTM reactions require participation of specific enzymes that add or remove particular functional groups to the appropriate protein substrates. When these enzymes operate near saturation with respect to their substrates, nonlinear signaling may occur, where input signals are amplified and switch output signals on or off [16,17]. When the protein substrates in a CMC are in excess relative to the modification or demodification enzymes, the degree of saturation of these enzymes depends on the Michaelis constants and the abundances of the contributing substrates.

The covalent modification status of a protein substrate not only modulates its activity, but may also alter its affinity as a substrate for the ubiquitination-proteasomal pathway that mediates the degradation of the majority of intracellular proteins [37]. Depending on whether the covalently modified protein molecule is a better or less suited substrate for ubiquitination, PTMs can either stabilize or destabilize a protein and thereby regulate its abundance. For instance, under normoxia, HIF-1α is oxidized by prolyl hydroxylase domain-containing proteins (PHD) in an oxygen-dependent manner and thereby targeted by the pVHL ubiquitination pathway for degradation, thus keeping the hypoxic transcriptional program under control [36,38]. As a different example, phosphorylation of p53 by ATM during the DNA damage response leads to its stabilization [1]. Therefore, the overall protein half-life and abundance do not remain constant in these situations, rather, they can change dynamically depending on the covalently modified fraction of the protein molecules. The altered protein substrate abundance in turn affects the degree of enzyme saturation, and hence creates an important nonlinearity in signaling.

A paradigm scenario of this type is PTM-induced protein stabilization on top of zero-order ultrasensitivity that pre-exists even for basal abundances of the protein substrates. In this scenario, as our simulations demonstrate, the degree of ultrasensitivity for the phosphorylated protein response (*R_p_*) with respect to the kinase *X* is considerably elevated, with *LRC* and the Hill coefficient increasing sharply as the half-life of *R_p_* is prolonged (Figure 3B,E). The enhancement of ultrasensitivity is due to the concurrently increased total protein substrate abundance as the input signal *X* increases, which pushes the kinase and phosphatase further into a saturated mode of operation. When the protein substrate is not high enough to enable zero-order ultrasensitivity at the basal condition, the increased protein substrate abundance induced by PTM can move the signaling motif toward saturation, thereby causing the emergence of ultrasensitivity, as demonstrated in Figure 5 and Figure S1. During the process of PTM-induced protein stabilization, the unmodified protein response is also enhanced for ultrasensitivity (Figure 3A,D) or rendered ultrasensitive (Figure 5A,D, Figures S1,D) although the response of *R* vs. *X* follows an inhibitory profile where *R* decreases as the input signal *X* increases.

An unexpected finding is the total protein response to the input signal (*R_tot_* vs. *X* for the MM model and free *R_tot_* vs. *X_tot_* for the full model), which can also exhibit ultrasensitivity, for both cases of PTM-induced protein stabilization and destabilization (Figure 3C, Figures S1C and S2C). The original Goldbeter–Koshland model was intended to examine either the covalently modified or unmodified protein responses under the condition of zero-order ultrasensitivity, while the total protein abundance stayed constant. Here, our simulations show that ultrasensitivity can emerge when there is an imbalance in the stability of the modified and unmodified proteins. When the modified protein is more stable, the total protein mass is dominated by the modified protein and thus resembles the modified protein response albeit with a non-zero basal level. By contrast, when the modified protein is less stable, the total protein response is dominated by the unmodified protein and thus resembles the unmodified protein response. In both situations, the response of *R_tot_* vs. *X* can be ultrasensitive because the modified or unmodified protein response is ultrasensitive. As an example, in the drosophila embryo, MAPK can phosphorylate transcriptional repressor Yan in response to morphogen gradients and thereby induce its degradation; this inducible degradation of Yan was proposed as part of a zero-order ultrasensitivity mechanism for the switch-like Yan response, which is responsible for the patterning of the embryonic ventral ectoderm [19]. Therefore, protein activity changes by PTM in a CMC are not mandatory for achieving zero-order ultrasensitivity if protein stability is also regulated by PTM. Using the full model, we also found that when there is a sufficient amount of demodifying enzyme to titrate the substrate, even under the condition that PTM results in protein stabilization, free total protein substrate *R_tot_* (*R* + *R_p_*) can display a mildly ultrasensitive, decreasing DR with respect to *X_tot_* (Figure 4C and Figure S2C). Moreover, if *R_p_* is further stabilized, free *R_tot_* exhibits nonmonotonic responses (Figure 4C), which further demonstrates that PTM-induced changes in protein stability can bring about more complex dose–response patterns. We also demonstrate that if the input-driving signal is supposed to increase the production rate of the protein substrate, a saturable covalent modification reaction, coupled with decreased stability of the modified protein, can also lead to an ultrasensitive increase in either the unmodified or total protein levels (Figure 8A,C, Figure S8A,C); this result confirms a recent finding by Mallela et al. [25]. However, we additionally found that the ultrasensitivity is enhanced in the full model and also applies to the modified protein (Figure S8). One uncertainty in our full model lies in the treatment of the stability of the protein substrates in complexes with their catalyzing enzymes. Since a protein in a multimeric protein complex can be selectively ubiquitinated and degraded, leaving the remaining components intact [30,31,32], it seems reasonable to assume that the protein substrate in complex with its enzyme is degraded with equal rate constant as the free substrate. It is of course possible that the stability of protein substrate in the complex can differ from that of its free form. The impact of this uncertainty is expected to be small if the substrate–enzyme complex is only a small fraction of the total protein substrate. However, if the fraction is significantly large, depending on the direction of the stability change, additional dynamics and DR response profiles may emerge, which will warrant further investigation.

In the absence of PTM-induced changes in protein stability, the CMC motif can launch a quick response amenable to the time scale associated with covalent modification reactions catalyzed by enzymes. However, when protein stability is altered by PTM with half-lives at the order of hours, it can take much longer for this signaling motif to reach a steady state (Figure 3G–I). If the protein substrate or its downstream target is a transcription factor, such as p53, HIF-1, BCL-6 or Yan, a relatively slow rise or activation may not matter much as far as the timeliness of a response is concerned, because the ensuing transcriptional induction of downstream genes takes much more time to complete anyway. Importantly, we propose here that ultrasensitivity through protein stabilization can be a potential energy-saving strategy employed by cells, where maintaining a high, saturating level of the protein substrate at basal condition may no longer be necessary. In addition, the initial overshoot exhibited by the *R* or *R_p_* response, as shown in Figure 3G,I, can also be a signaling strategy utilized by cells to accelerate transcriptional induction for gene production with long half-lives [39].

Throughout the result section and the Supplemental Materials, we have compared the degree of steepness of the steady-state DR curve as quantified by *n_H_* with the degree of true ultrasensitivity quantified by *LRC* and confirmed their known differences in describing ultrasensitive DR curves [12,34]. While the two metrics in most situations move in the same direction in response to changes in a parameter value, the corresponding |*n_H_*| for a particular DR curve can be higher or lower than |*LRC*|*_max_*. A higher |*n_H_*| value means an overestimate of the degree of amplification of the DR curve, which often occurs when the DR curve has a significant basal level. There are also scenarios where the DR curve exhibits a profile comprising of an almost linear response followed immediately by a plateau (Figure 8B and Figure S5B). Such a response profile may have an apparent *n_H_* = 2 despite the fact that its response is at most linear. We have also encountered DR curves having an |*LRC*|*_max_* value higher than |*n_H_*| (Figure S6B,C); in these situations, *n_H_* underestimates the degree of amplification. Therefore, when examining the ultrasensitivity of a DR curve, it is advisable to quantify both *LRC* and *n_H_*.

Building upon Goldbeter and Koshland’s concepts, Mallela et al. recently proposed mathematical models for protein modification cycles, focusing, in particular, on protein substrates that are ubiquitinated by the same E3 ligases, which mark both proteins for degradation [25]. Many E3 ligases are apparently promiscuous, thereby permitting competition between “similar” protein substrates. The authors observed that the sensitivity to incoming signals, as well as the ultrasensitivity of the response, is diminished or even destroyed when the protein substrate saturates the modifying enzyme. This ultrasensitivity-weakening effect is more dramatic if the cycling proteins are degraded at a relatively high rate, consistent with our earlier findings [24]. However, even though the authors used the full model, their study only considered the situation when the total protein substrate is at least 1000-fold higher than the demodifying enzyme, while in the present study we systemically examined the condition when these two quantities are comparable or even when the enzyme is at a higher level. Mallela and colleagues also found that signaling cycles, in which the coupling of protein substrates collectively leads to saturation of the enzymes, can lead to a coupled, switch-like response in all protein substrates, likely due to the competition or “crosstalk” of the substrate proteins with respect to the same E3 ligases. The effects of protein turnover on ultrasensitivity does not seem to be limited to the CMC motif explored here. It also plays a modulatory role in the ultrasensitivity arising from molecular titration or protein sequestration [40].

The signaling motif of a CMC can exhibit complex dynamic behaviors and has been extensively studied with computational means. Wang et al. investigated and decomposed the tunability of the zero-order ultrasensitivity [41]. Xu and Gunawardena examined some more realistic intracellular situations where multiple enzyme intermediates exist due to co-substrate binding for both reversible and irreversible reactions and found that these complications modulate the zero-order switching behavior [42]. The operation of the CMC in the face of protein expression noise has been explored more recently [43,44]. It seems important to have correlated expression of the paired modification and demodification enzymes to prevent switch flipping, and bifunctional enzymes in a CMC may be an ideal solution in this regard [44]. Using linear reactions of the modification and demodification reactions, Soyer demonstrated that the CMC motif, like negative feedback or incoherent feedforward loops, can exhibit transient or persistent dynamic responses depending on the difference in protein stability [45]. In the present study, we have added a new aspect by demonstrating that PTM-associated changes in protein stability, enzyme features, or protein synthesis offer the cell another level of sophistication regarding the complex response behaviors of this long-studied signaling motif.

## Figures and Tables

**Table 1:** Default parameter values for the MM model in Figure 1C.

Parameter	Description	Default Value
*k*_0_	Rate constant of synthesis of *R*	1 (concentration/time)
*k*_1_	Catalytic rate constant for phosphorylation	10 (1/time)
*K_m_*_1_	Michaelis constant for phosphorylation	10 (concentration)
*X*	Kinase	0 (concentration)
*k*_2_	Catalytic rate constant for dephosphorylation	10 (1/time)
*K_m_*_2_	Michaelis constant for dephosphorylation	10 (concentration)
*k*_3_	Degradation rate constant of *R*	0.01 (1/time)
*k*_4_	Degradation rate constant of *R_p_*	0.01 (1/time)
*Y*	Phosphatase	1 (concentration)

**Table 2:** Default parameter values for the full model in Figure 1D.

Parameter	Description	Default Value
*k*_0_	Rate constant of synthesis of *R*	1 (concentration/time)
*k*_1f_	Association rate constant for *R* and *X* binding	10 (1/concentration/time)
*k*_1b_	Dissociation rate constant for *RX* complex	90 (1/time)
*k*_1c_	Catalytic rate constant for phosphorylation	10 (1/time)
*X_tot_*	Total kinase	0 (concentration)
*k*_2f_	Association rate constant for *R_p_* and *Y* binding	10 (1/concentration/time)
*k*_2b_	Dissociation rate constant for *R*_p_*Y* complex	90 (1/time)
*k*_2c_	Catalytic rate constant for dephosphorylation	10 (1/time)
*k*_3_	Degradation rate constant of *R* and *RX*	0.01 (1/time)
*k*_4_	Degradation rate constant of *R_p_* and *R_p_Y*	0.01 (1/time)
*Y_tot_*	Total phosphatase	1 (concentration)

**Table 3:** Summary of effects of varying parameters on ultrasensitivity.

Parameter Varied	Stability Condition	Effects on |*LRC*|*_max_*		
		*R* vs. *X_tot_*	*R_p_* vs. *X_tot_*	*R + R_p_* vs. *X_tot_*
↓*k*_4_	*k*_3_ > *k*_4_	↑#, – *	↑	↑#, ↓ *
↑*k*_4_	*k*_3_ < *k*_4_	↓#, – *	↓	↑
↓*K_m_*_1_, ↓*K_m_*_2_	*k*_3_ > *k*_4_ or *k*_3_ < *k*_4_	↑	↑	↑
↑*K_m_*_1_, ↑*K_m_*_2_	*k*_3_ > *k*_4_ or *k*_3_ < *k*_4_	↓	↓	↓
↑*k*_1_, ↑*k*_21_	*k*_3_ > *k*_4_ or *k*_3_ < *k*_4_	–	–	–
↓*k*_1_, ↓*k*_21_	*k*_3_ > *k*_4_ or *k*_3_ < *k*_4_	–	–	–
↑*k*_2_, ↑*k*_2*c*_	*k*_3_ > *k*_4_	↑	↑	↑
↑*k*_2_, ↑*k*_2*c*_	*k*_3_ < *k*_4_	–	–	–
		*R* vs. *k*_0_	*R_p_* vs. *k*_0_	*R + R_p_* vs. *k*_0_
↓*k*_4_	*k*_3_ > *k*_4_	–	–	–
↑*k*_4_	*k*_3_ < *k*_4_	↑	–	↑

Note: ↑, ↓, and – denote increase, decrease, and no/very small effect, respectively. Unless otherwise indicated, the effects apply to both MM and full models. # Change that occurs with MM model only. * Change that occurs with full model only.

## Supplementary Materials

The following are available online at https://www.mdpi.com/article/10.3390/biom11111741/s1, Figures S1–S8, MATLAB and R model code.
