# Supplementary material for: Emergence and Enhancement of Ultrasensitivity through Posttranslational Modulation of Protein Stability
Source: Biomolecules. 2021 Nov 22;11(11):1741. doi: 10.3390/biom11111741 (PMC8615576; doi:10.3390/biom11111741)
Supplement: Supplementary file 1 [file biomolecules-11-01741-s001.zip › Supplemental Materials.pdf]

Supplemental Material

Emergence and Enhancement of Ultrasensitivity through Posttranslational Modulation of Protein Stability

Carla M. Kumbale, Eberhard O. Voit, and Qiang Zhang

Ordinary differential equations for the full model

$$\frac{dR}{dt} = k_0 - k_3 * R - k_{1f} * X * R + k_{1b} * RX + k_{2c} * R_pY, \tag{S1}$$

$$\frac{dR_p}{dt} = k_{1c} * RX - k_4 * R_p - k_{2f} * R_p * Y + k_{2b} * R_pY, \tag{S2}$$

$$\frac{dRX}{dt} = k_{1f} * X * R - k_{1b} * RX - k_{1c} * RX - k_3 * RX, \tag{S3}$$

$$\frac{dR_pY}{dt} = k_{2f} * R_p * Y - k_{2b} * R_pY - k_{2c} * R_pY - k_4 * R_pY, \tag{S4}$$

where $X = X_{tot} - RX$ and $Y = Y_{tot} - R_pY$.

Additional Figures and Explanations

1. Emergence of ultrasensitivity in the full model

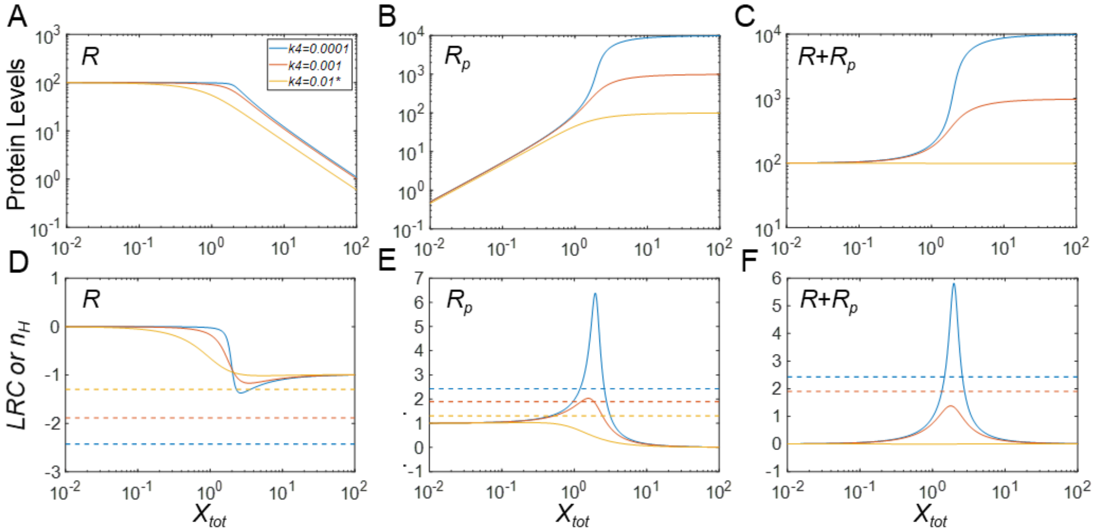

Figure S1. Emergence of ultrasensitivity through phosphorylation-induced protein stabilization in the full model (with increased apparent K_m values). (A-C) Steady-state DR curves for R *vs.* X_{tot}, R_p *vs.* X_{tot}, and free R_{tot} (R+R_p) *vs.* X_{tot}, respectively, for different values of k_4, as indicated in panel A. The same color-scheme for k_4 values holds for all panels. As k_4 decreases, ultrasensitivity emerges for R_p and free R_{tot}. **(D-F)** *LRC* (solid lines) and n_H (dashed horizontal lines) for R, R_p, and free R_{tot}, respectively, for different values of k_4. *k_4=0.01 is the default value. For these simulations, k_{1b}=k_{2b}=990 such that the apparent K_{m1}=k_{m2}=100.

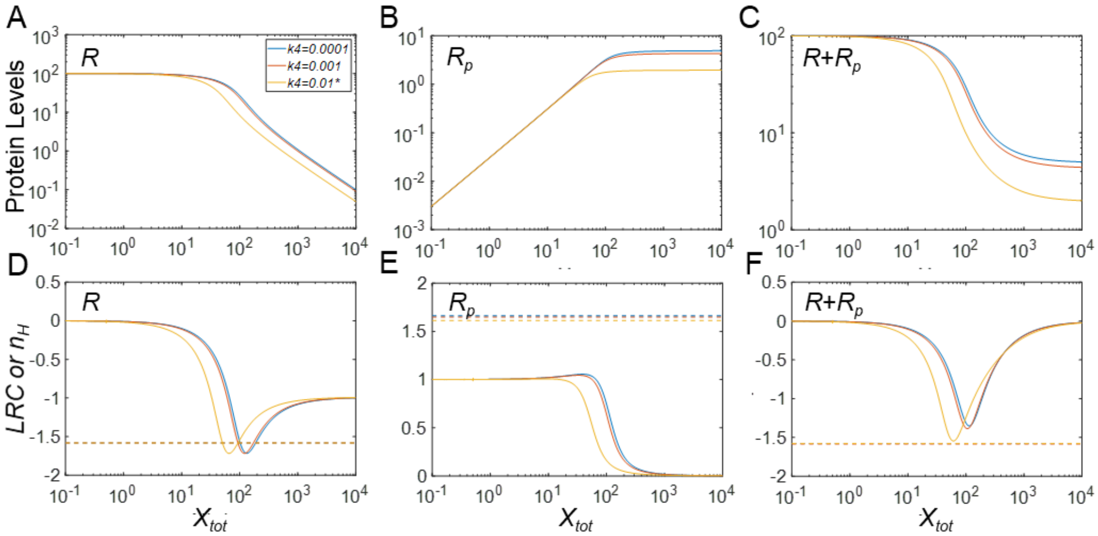

Figure S2. Emergence of ultrasensitivity through phosphorylation-induced protein stabilization in the full model (with increased enzyme *Y* level). (A-C) Steady-state DR curves for R *vs.* X_{tot}, R_p *vs.* X_{tot}, and free R_{tot} (R+R_p) *vs.* X_{tot}, respectively, for different values of k_4, as indicated in panel A. The same color-scheme for k_4 values holds for all panels. As k_4 decreases, ultrasensitivity emerges for R_p and free R_{tot}. **(D-F)** *LRC* (solid lines) and n_H (dashed horizontal lines) for R, R_p, and free R_{tot}, respectively, for different values of k_4. *k_4=0.01 is the default value. For these simulations, Y_{tot}=300.

2. *Effects of Michaelis constant K_{m2}*

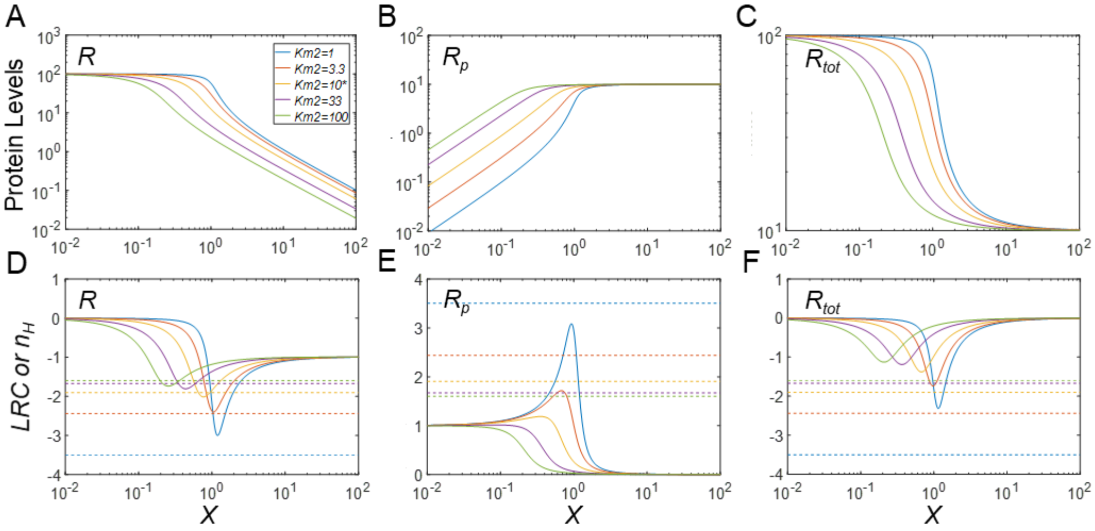

Figure S3. Effects of K_{m2} on ultrasensitivity with phosphorylation-induced protein destabilization in the MM model (k_4 = 0.1). (A-C) Steady-state DR curves for R *vs.* X, R_p *vs.* X, and R_{tot} *vs.* X, respectively, for different values of K_{m2}, as indicated in panel A. The same color-scheme for K_{m2} is used for all panels. **(D-F)** *LRC* (solid lines) and n_H (dashed horizontal lines) for R, R_p, and R_{tot}, respectively. * K_{m2}=10 is the default value.

In the analysis leading to Fig. S3, we explore the role of the Michaelis constant K_{m2} and examine the case where phosphorylation of R to R_p results in destabilization; as the baseline, we set k_4=0.1, which is 10-fold greater than k_3. As K_{m2} decreases, the DR curves for R, R_p, and R_{tot} become increasingly steeper (Figs. S3A-S3C). For low K_{m2} values, $|LRC|_{max}$ can be much greater than $|n_H|$, whereas for high K_{m1} values, $|LRC|_{max}$ approaches 1 for R_p, indicating loss of ultrasensitivity (Figs. S3B and S3E). For the R response, increasing K_{m1} reduces the steepness of the DR curve without complete loss of ultrasensitivity (Figs. S3A and S3D). Lastly, increasing K_{m1} reduces the steepness of the DR curve for R_{tot} with $|n_H|$ approaching a fixed value slightly below 2, and ultrasensitivity is lost for high K_{m1} values as indicated by $|LRC|$ (Figs. S3C and S3F).

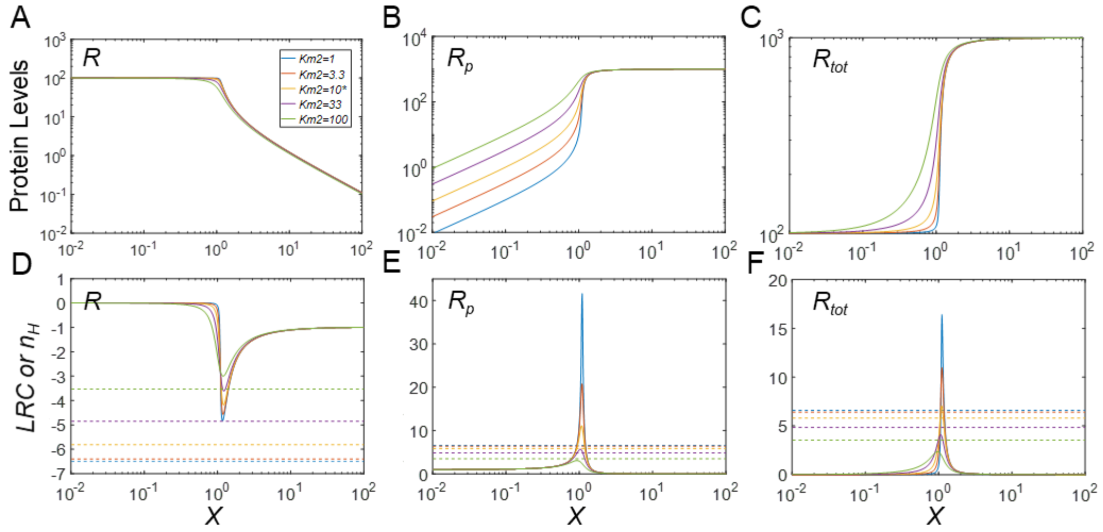

Figure S4. Effects of K_{m2} on ultrasensitivity with phosphorylation-induced protein stabilization in the MM model (k_4 = 0.001). (A-C) Steady-state DR curves for R *vs.* X, R_p *vs.* X, and R_{tot} *vs.* X, respectively, for different values of K_{m2}, as indicated in panel A. The same color-scheme for K_{m2} is used for all panels. **(D-F)** *LRC* (solid lines) and n_H (dashed horizontal lines) for R, R_p, and R_{tot}, respectively. * K_{m2}=10 is the default value.

The rationale for the second analysis is a situation where phosphorylation of R into R_p results in strong protein stabilization (k_4=0.001, 10-fold lower than k_3). When K_{m2} decreases below its baseline value of 10, the DR curves for R_p and R_{tot} become increasingly steeper with minimal changes in the steepness for R (Fig. S4A-S4C). For the response of R, $|n_H|$ obviously overestimates the degree of ultrasensitivity as evaluated by $|LRC|_{max}$ (Fig. S4A and S4D). For the R_p response, increasing K_{m2} reduces the steepness of the DR curve with $|LRC|_{max}$ consistently greater than $|n_H|$ (Fig. S4B and S4E). Lastly, increasing K_{m1} reduces the steepness of the DR curve for R_p with $|n_H|$ overestimating the degree of ultrasensitivity as represented by $|\text{LRC}|_{\text{max}}$ for large K_{m2} values, and underestimating for small K_{m2} values (Fig. S4C and S4F).

3. Effects of catalytic constant k_2

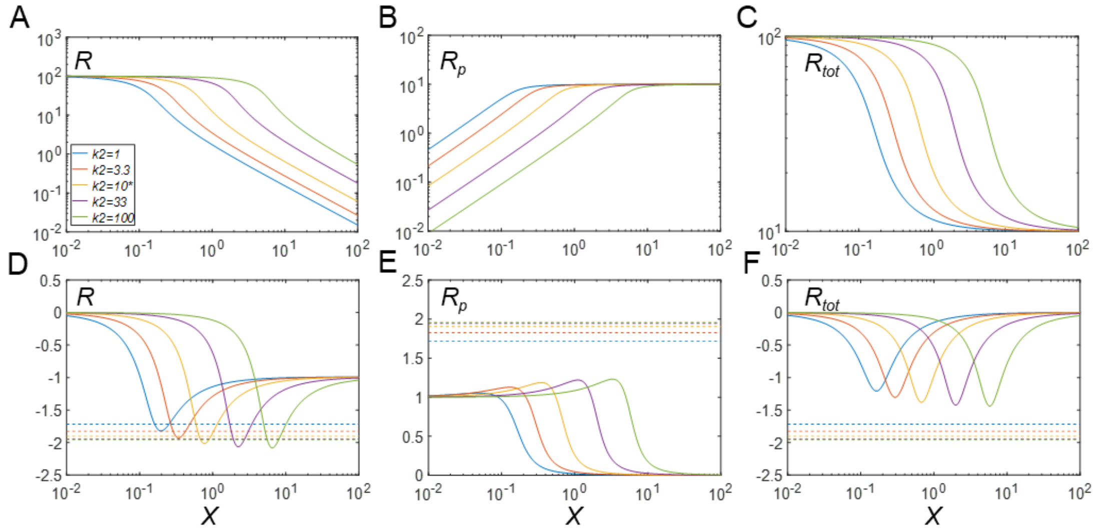

Figure S5. Effects of k_2 on ultrasensitivity with phosphorylation-induced protein destabilization (k_4 = 0.1) in the MM model. (A-C) Steady-state DR curves for R *vs.* X, R_p *vs.* X, and R_{tot} *vs.* X, respectively, for different values of k_2, as indicated in panel A. The same color-scheme for k_2 is used for all panels. **(D-F)** *LRC* (solid lines) and n_H (dashed horizontal lines) for R, R_p, and R_{tot}, respectively. * k_2=10 is the default value.

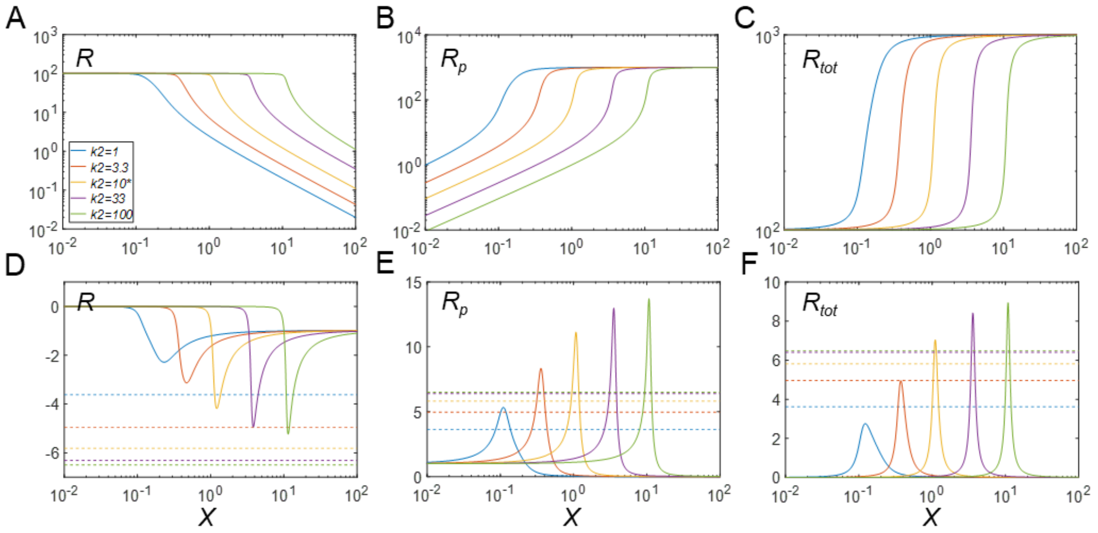

Figure S6. Effects of k_2 on ultrasensitivity with phosphorylation-induced protein stabilization in the MM model (k_4 = 0.001). (A-C) Steady-state DR curves for R *vs.* X, R_p *vs.* X, and R_{tot} *vs.* X, respectively,

for different values of k_2, as indicated in panel A. The same color-scheme for k_2 values is used for all panels. **(D-F)** *LRC* (solid lines) and n_H (dashed horizontal lines) for R, R_p, and R_{tot}, respectively. * k_2=10 is the default value.

Instead of focusing on alterations in K_m values, one may explore here the effects of varying k_2, the catalytic rate of the dephosphorylation step, on ultrasensitive behavior when changes in protein stability are induced by PTMs. We first examine the case where phosphorylation of R to R_p results in destabilization (k_4=0.1, 10-fold greater than k_3). As shown in Fig. S5, k_2 only has a marginal effect on the degree of ultrasensitivity as assessed by either n_H or *LRC*, while decreasing k_2 generally sensitizes all responses, with the DR curves shifting to the left. Increasing k_2 appears to increase $|n_H|$ and $|LRC|_{max}$ only slightly, and all DR curves approach a fixed value when k_2 becomes very large. For the case where phosphorylation of R into R_p results in protein stabilization (k_4=0.001, 10-fold lower than k_3), as shown in Fig. S6, increasing k_2 desensitizes all responses and shifts the DR curves to the right, thereby increasing the degree of ultrasensitivity. However, for very high k_2 values, both $|n_H|$ and $|LRC|_{max}$ seem to reach some maximal values.

4. *Ultrasensitivity in response to changes in protein synthesis*

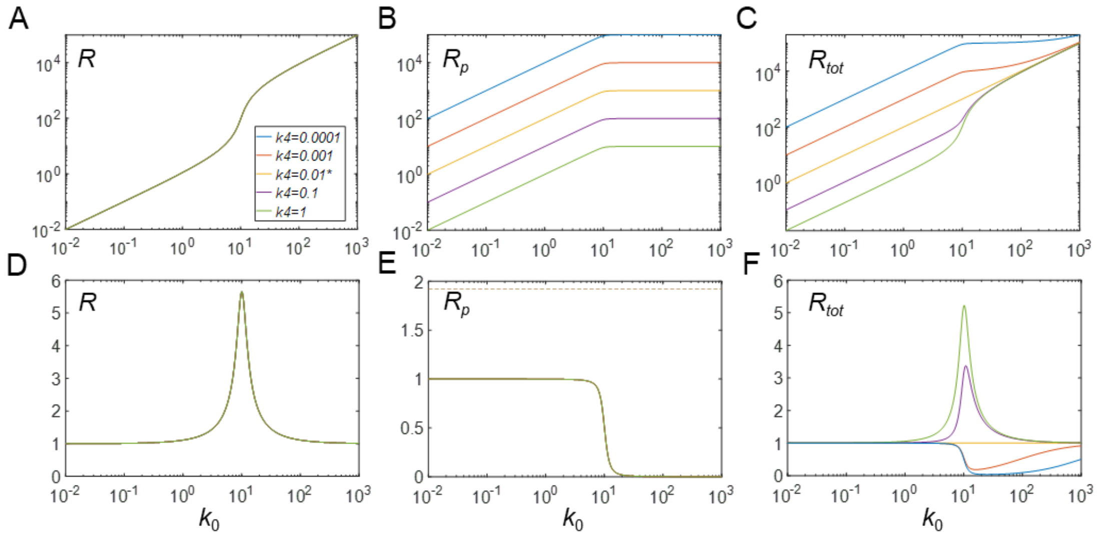

Figure S7. k_0-driven ultrasensitivity with phosphorylation-induced changes in protein stability in the MM model (k_2=0). (A-C) Steady-state DR curves for R *vs.* k_0, R_p *v.s* k_0, and R_{tot} *vs.* k_0, respectively, for different values of k_4, as indicated in A. The same color-scheme for k_4 values is used for all panels. **(D-F)** *LRC* (solid lines) and n_H (dashed horizontal lines) for R, R_p, and R_{tot}. * k_4=0.01 is the default value. X=1 for all conditions. Note that no n_H was evaluated for R and R_{tot} because the responses do not saturate. Note: in panels A, D and E, all five curves overlap.

To further explore the mechanism of ultrasensitivity driven by the synthesis rate k_0, the dephosphorylation step is disabled by setting k_2=0. It is clear for the DR curve of R vs k_0 that ultrasensitivity remains regardless the direction in which phosphorylation alters the stability of R_p (Figs. S7A and S7D), suggesting that it is the saturation of the phosphorylation that is responsible for the ultrasensitivity. For the DR curve of R_{tot} vs k_0, ultrasensitivity is only present when k_4>k_3, *i.e.*, phosphorylation results in destabilization of R_p (Figs. S7C and S7E), where R dominates the fraction of R_{tot}. No ultrasensitivity exists for R_p (Figs. S7C and S7F).

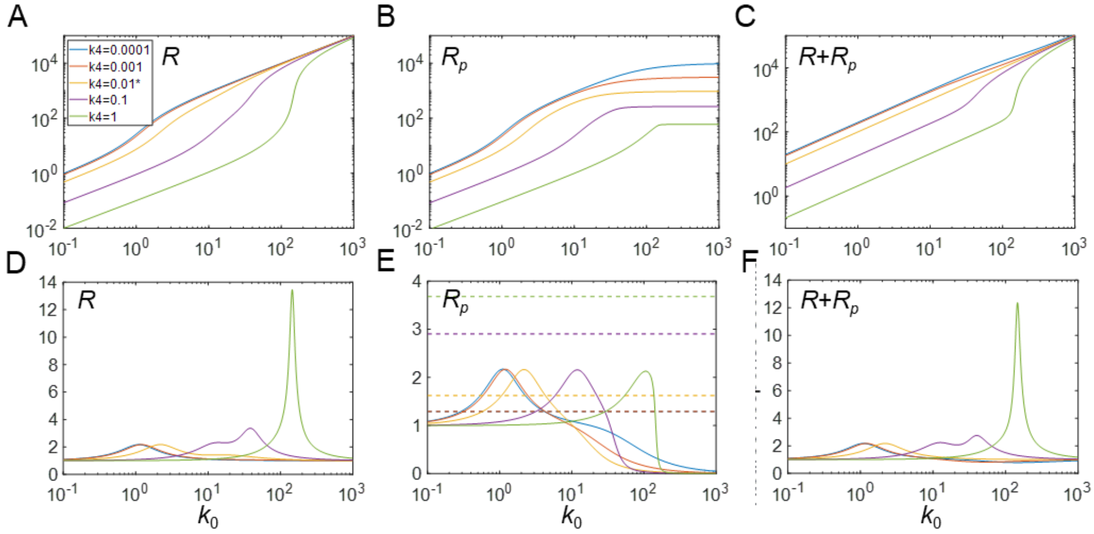

Figure S8. k_0-driven ultrasensitivity with phosphorylation-induced changes in protein stability in the full model (X_{tot} = Y_{tot} = 100). (A-C) Steady-state DR curves for R *vs.* k_0, R_p *v.s* k_0, and free R_{tot} (R+R_p) *vs.* k_0, respectively, for different values of k_4, as indicated in (A). The same color-scheme for k_4 values is used

for the other panels. **(D-F)** *LRC* (solid lines) and n_H (dashed horizontal lines) for R, R_p, and free R_{tot}. * k_4=0.01 is the default value. Note that no n_H was evaluated for R and R_{tot} because the responses do not saturate.
